# An in vivo electrophysiological preparation for mechanical, electrical and optical stimulation of sensory neurons that innervate murine bone

**DOI:** 10.1002/ame2.70097

**Published:** 2025-10-23

**Authors:** Michael Morgan, Hoi Ying Lee, Aung Aung Kywe Moe, Jenny Thai, Jackson Hart, Jason J. Ivanusic

**Affiliations:** ^1^ Department of Anatomy and Physiology University of Melbourne Melbourne Victoria Australia; ^2^ Present address: Department of Medical Imaging and Radiation Sciences, School of Primary and Allied Health Care Monash University Melbourne Victoria Australia

**Keywords:** bone, electrophysiology, nerve, optogenetic, pain, skeletal

## Abstract

In this study, we aimed to develop an in vivo electrophysiological bone‐nerve preparation to record the activity of peripheral sensory neurons that innervate the murine tibia. A small nerve that innervates the tibial marrow cavity was identified in isoflurane‐anesthetized C57BL/6 mice, and placed over a platinum hook electrode for extracellular recording. Whole‐nerve activity was amplified, filtered and sampled at 20 kHz using PowerLab (ADInstruments). A cannula was placed into the marrow cavity to deliver mechanical stimuli (by pressurizing with injection of saline) and/or capsaicin. Optical stimulation was achieved by application of 473 nm blue light (1 Hz, 0.25–0.5 ms, 0–12.5 mW/mm) to the tibial marrow cavity in *Wnt1‐Cre; loxP‐ChR2* mice. Murine bone afferent neurons responded to high threshold noxious mechanical stimulation, coded for the intensity of mechanical stimulation, could be sensitized by capsaicin, and did not suffer stimulus‐evoked fatigue at 10‐minute interstimulus intervals. Electrical and optical stimulation within the marrow cavity evoked action potentials with conduction velocities in the Aδ and/or C fiber range. These new approaches to recording the activity of bone afferent neurons will allow us to take advantage of transgenic and optogenetic tools to further our understanding of mechanisms that generate and maintain bone pain in the future.

## Graphical abstract

Recording the activity of sensory neurons that innervate bone in response to optical, electrical and mechanical stimulation.

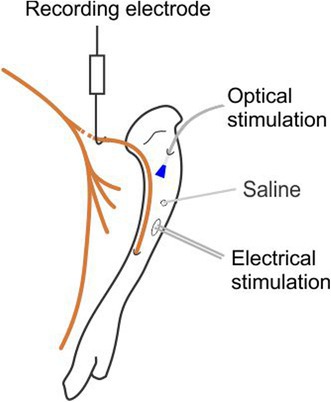

## 1 INTRODUCTION

The development of new strategies to treat skeletal pain has been hindered by a lack of appropriate research tools to identify mechanisms that generate and maintain pain of skeletal origin. To address this, our laboratory has previously developed an in vivo electrophysiological bone‐nerve preparation to record the activity and sensitivity of single sensory neurons innervating the tibial marrow cavity (bone afferent neurons) of the rat.^1^, ^2^ Using this approach, we have demonstrated how bone afferent neurons respond to noxious mechanical stimuli, algesic substances and inflammatory mediators,^1^, ^2^, ^3^, ^4^, ^5^, ^6^ identified roles for a number of different ligands, ion channels and receptors in their function,^5^, ^7^, ^8^ and have studied their contribution to pain in conditions such as osteoarthritis.^9^, ^10^, ^11^ However, this rat preparation relies mostly on pharmacological manipulation to probe the roles of specific neurons and signaling pathways. In the present study, we aimed to develop and characterize a murine preparation to record from bone afferent neurons, to better leverage the vast array of powerful transgenic tools, including optogenetics, that have emerged to more precisely interrogate the functions of genes and neurons involved in pain processing and pathology.^12^

## 2 METHODS

Here we present an overview of the methodology used in the present study. A full description of the methodology, including source of animals, equipment and reagents, is provided in Supplementary Methods.

**FIGURE 1:**
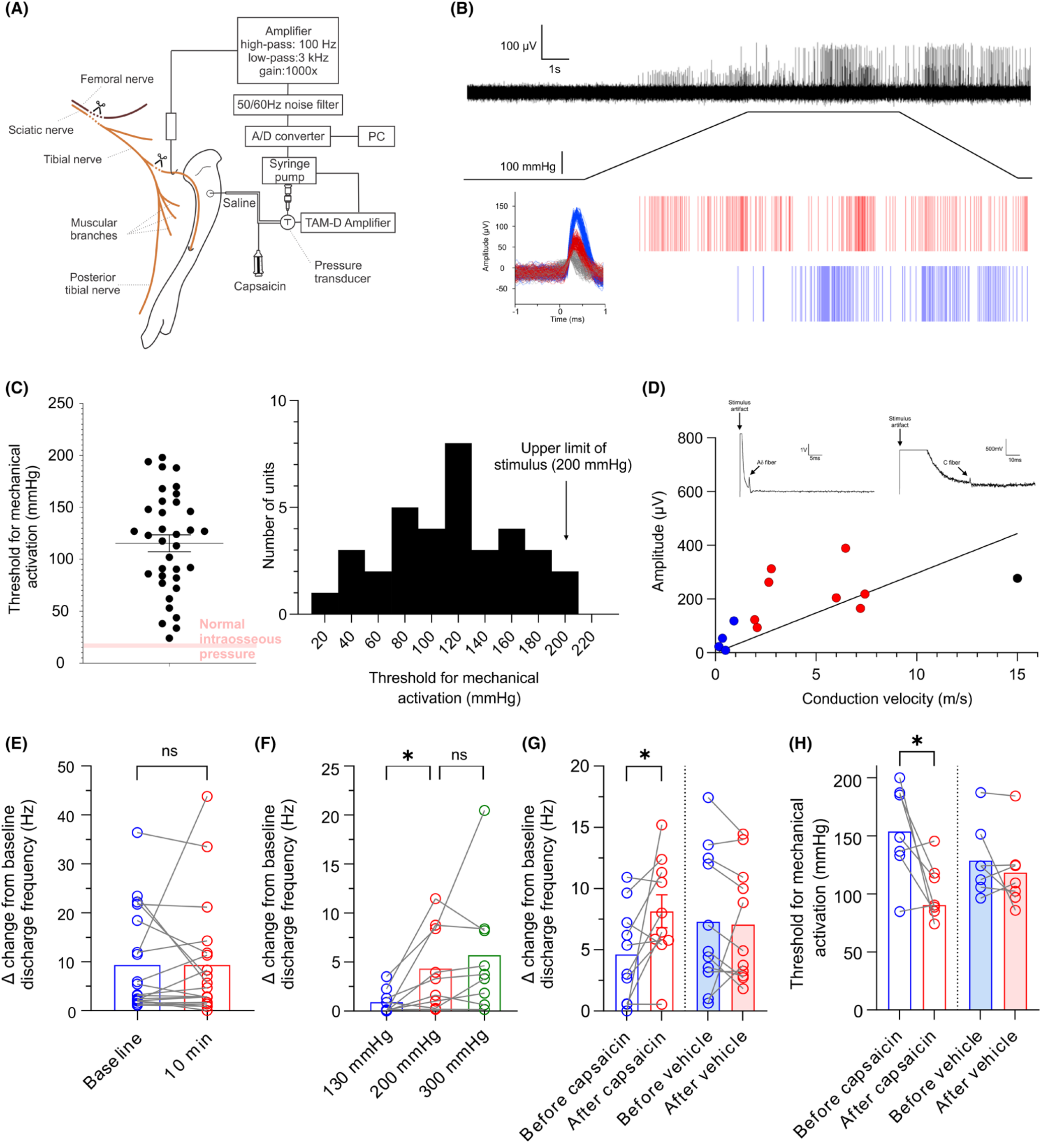
Mechanical and electrical stimulation of murine bone afferent neurons. (A) Schematic diagram of the experimental protocol to perform electrophysiological recording from bone afferent neurons in mice. (B) An example whole‐nerve recording (top) and rasters of single unit activity (bottom) in response to mechanical stimulation of the marrow cavity with a 300 mmHg ramp‐and‐hold pressure stimulus (middle). Single units were isolated by similarities in spike duration and amplitude (inset). In this example, each of the isolated units had a clear response to increased intraosseous pressure during the ramp phase of the stimulus, and a sustained response through both the hold phase and for some time after the stimulus. (C) The thresholds for mechanical activation of single bone afferent neurons were between 24 and 198 mmHg (126.4 ± 7.4 mmHg; *N* = 21, *n* = 35). The distribution of thresholds for mechanical activation was unimodal, with >85% of units having thresholds 5 times that of normal intraosseous pressure (16–19 mmHg). (D) Electrical stimulation of the marrow cavity resulted in activation of neurons with conduction velocities predominantly in the Aδ and C fiber range (0.17–15 m/s; *N* = 8, *n* = 13). Insets show representative examples of whole‐nerve recordings in response to electrical stimulation. Single units with C fiber conduction velocities typically had spike amplitudes less than 65 μV (blue circles), whereas single units with Aδ fiber conduction velocities typically had spike amplitudes greater than 75 μV (red circles). There was a linear relationship between conduction velocity and amplitude of single units (slope of line = 29.57). (E) Single unit discharge frequencies in response to a 200 mmHg pressure stimulus showed no difference between baseline responses and after a 10‐minute ISI (paired *t* test, *p* = 0.9983; *N* = 6, *n* = 19). (F) A significant increase in the discharge frequency was observed in response to increased pressure (one‐way ANOVA with repeated measures; *F*(1.681, 13.45) = 5.506, *p* = 0.0217; *N* = 3, *n* = 9), with an increase in single unit discharge frequency from 130 to 200 mmHg (Bonferroni's post hoc test, *p* = 0.043), but not between 200 and 300 mmHg (Bonferroni's post hoc test, *p* = 0.738). (G) Administration of capsaicin resulted in a significant increase in single unit discharge frequency in response to mechanical stimulation (paired *t* test, *p* = 0.0476; *N* = 5, *n* = 10), but there was no change in mechanically evoked single unit discharge frequency after administration of vehicle (paired *t* test, *p* = 0.814; *N* = 4, *n* = 11). (H) Administration of capsaicin resulted in a significant decrease in single unit threshold for mechanical activation (paired *t* test, *p* = 0.035; *N* = 4, *n* = 7), but there was no change in threshold for mechanical activation after administration of vehicle (paired *t* test, *p* = 0.358; *N* = 4, *n* = 7).

C57BL/6 mice were anesthetized with isoflurane and prepared for electrophysiological recording. A fine branch of the nerve innervating the marrow cavity of the murine tibia was exposed, cut and placed onto a platinum hook electrode (Figure 1A). The nerve to the tibia and the sciatic and femoral nerves were transected to prevent reflex activation of muscle or sympathetic efferent neurons (Figure 1A). Whole‐nerve electrical activity was amplified, filtered, sampled and stored to PC (Figure 1A). Mechanical stimuli were delivered by increasing tibial intraosseous pressure using a feedback‐controlled syringe pump (Figure 1A), to deliver a ramp‐and‐hold mechanical stimulus with an initial flow rate of 2 mL/min (ramp phase), and a constant pressure of 5 s duration (hold phase) (Figure 1B). Nerve impulses arising from single, mechanically or electrically activated units were discriminated from the whole‐nerve recordings using Spike Histogram software (Figure 1B). Threshold for mechanical activation was determined as the pressure at which each unit started firing, or increased firing, during the ramp phase. Discharge frequency was expressed as the number of spikes per second (Hz), determined over the entire ramp‐and‐hold stimulus, and was normalized by reporting it as the difference between a 5 s period before and during the pressure stimulus. Conduction velocity was determined by electrical stimulation with bipolar silver electrodes (0.5 Hz, 0.02–2 ms pulse duration, 0.05–10 V) placed directly into the marrow cavity through small holes made in the bone. Waveform average analysis was used to identify activity time‐locked to electrical stimulation (10–100 sweeps averaged, 200 ms pre‐trigger, 800 ms post‐trigger). Aδ fibers were classified using conduction velocities between 1.4 and 13.6 m/s, while C fibers were slower than 1.4 m/s.^13^

**FIGURE 2:**
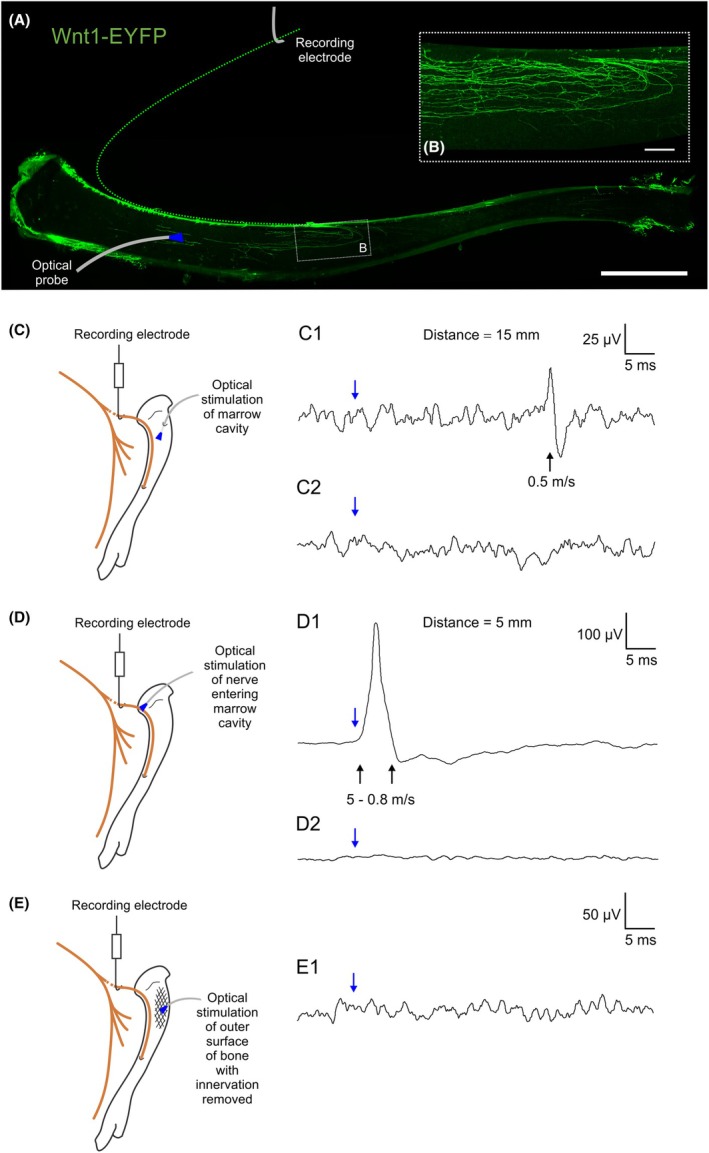
Optical stimulation of murine bone afferent neurons. (A) Whole‐tissue immunolabeling and 3D fluorescence imaging to visualize EYFP positive nerve fibers and supporting cells in the tibia of *Wnt1‐ChR2* mice. A 150 μm maximum intensity z‐projection showing bone marrow and cortical bone (scale bar = 2000 μm). The distance used to calculate conduction velocity was estimated as the length between the recording electrode and nutrient foramen where it entered the bone, combined with the distance between the nutrient foramen and the site of optical stimulation in the tibial marrow cavity. (B) Inset shows a 1000 μm maximum intensity *z*‐projection of EYFP‐labeled nerve fibers and/or supporting cells (scale bar = 200 μm). (C) Optical stimulation within the marrow cavity of the tibia resulted in compound action potentials in *Wnt1‐ChR2* mice (C1; *N* = 3), but not in C57BL/6 control mice (C2; *N* = 4). (D) Optical stimulation of the nerve to the tibia before it entered the marrow cavity also resulted in compound action potentials in *Wnt1‐ChR2* mice (D1; *N* = 4), but not C57BL/6 control mice (D2; *N* = 4). (E) Optical stimulation of the outer surface of the cortical bone of *Wnt1‐ChR2* mice failed to elicit any measurable response (E1; *N* = 3). In C to E, each trace is an average of 100 sweeps, blue arrows show time of optical stimulus onset, and conduction velocities were estimated using the distance between the optical stimulus probe and the recording electrode, reported above each trace.

We used tissue clearing and light sheet microscopy to confirm expression of ChR2 in nerve terminal endings of bone afferent neurons in the marrow cavity of *Wnt1‐ChR2* mouse tibia (see Supplementary Methods)^14^. To optically stimulate bone afferent neurons, *Wnt1‐ChR2* and C57BL/6 mice were anesthetized and prepared for electrophysiological recording as above. A 473 nm laser probe was placed into the tibial marrow cavity through a small hole made through the cortex (Figure 2A,C). Optical stimulation was also performed with the laser illuminating the nerve to the tibia before it entered the marrow cavity (Figure 2D), and on the outer surface of the cortical bone with periosteum scraped away (Figure 2E). Laser powers of 0–12.5 mW/mm were delivered at 1 Hz, using the shortest laser pulse duration (0.25–0.5 ms) that produced reliable changes in activity. Whole‐nerve electrical activity was recorded as above. Waveform average analysis was used to identify activity time‐locked to optical stimulation (100 sweep average, 20 ms pre‐trigger, 110 ms post‐trigger). Conduction velocity was estimated from the distance of the nerve path between the recording electrodes and the optical probe tip (Figure 2A).

Statistical analyses were performed using GraphPad Prism (version 10.4.1, Boston, MA, USA).

## 3 RESULTS

Mechanical stimulation of the marrow cavity resulted in increased activity in the nerve to the murine tibia (Figure 1B). Single units were isolated by spike discrimination based on action potential duration and amplitude (Figure 1B). The threshold for mechanical activation of single units was between 24 and 198 mmHg (Figure 1C). Electrical stimulation of the bone marrow revealed that murine single bone afferent neurons had conduction velocities consistent with Aδ and C fiber classifications (Figure 1D). There was no difference in the discharge frequency of single units in response to repeated mechanical stimulation with pairs of 200 mmHg ramp‐and‐hold stimuli delivered at a 10‐minute ISI (Figure 1E), indicating no stimulus evoked fatigue over this time interval. To determine whether bone afferent neurons code for the intensity of mechanical stimulation, we compared single unit responses to a series of pressure stimuli (130, 200 and 300 mmHg), delivered in randomized orders. Increasing the intensity of mechanical stimulation of the marrow cavity resulted in increased discharge frequency (Figure 1F). To determine if bone afferent neurons can be sensitized to mechanical stimulation, their discharge frequency and threshold for activation were assessed in response to a 200 mmHg ramp‐and‐hold pressure stimulus, delivered before and 30 minutes after application of capsaicin (2 μmol/L, 10 μL) or vehicle (saline, 10 μL). Application of capsaicin, but not vehicle, to the endings of bone afferent neurons sensitized them to mechanical stimulation (Figure 1G,H).

Extensive immunolabeling of the reporter protein EYFP was observed in marrow cavity axons and peripheral nerve terminal endings of *Wnt1‐ChR2* mice (Figure 2A,B). Optical stimulation of the marrow cavity of *Wnt1‐ChR2* mice reliably induced compound action potentials when the stimulus intensity was 2.5 mW/mm^2^ and pulse duration was 0.3 ms or greater (Figure 2C). Conduction velocities suggested contributions predominantly from C fibers. In some cases we did observe responses attributable to Aδ fibers, but these were a lot less consistent and robust and are thus not reported further. The same stimulation protocol failed to evoke any responses when delivered to the tibial marrow cavity of C57BL/6 mice (Figure 2C). Optical stimulation of the nerve before it enters the tibia also produced reliable compound action potentials in *Wnt1‐ChR2*, but not C57BL/6 mice (Figure 2D). Optical stimulation on the outside of the bone of *Wnt1‐ChR2* mice, after all periosteal afferents were scraped away from the surface of the cortical bone, did not elicit a compound action potential at the maximum stimulus intensity (12.5 mW/mm^2^) and pulse duration (5 ms) (Figure 2E). This confirms that the optical stimuli we applied were unable to penetrate bone, and provides a unique opportunity to localize optical stimuli to afferent neurons selectively innervating the bone marrow.

## 4 DISCUSSION

This preparation allows us to record the activity of bone afferent neurons in response to noxious mechanical, electrical and optical stimuli applied to the tibial marrow cavity of mice, and provides a unique opportunity to use transgenic animals to study the role of bone afferent neurons in generating and maintaining bone pain in the future. Importantly, the functional properties of murine bone afferent neurons we have reported here are the same as that previously reported using our rat bone‐nerve preparation.^1^, ^2^, ^3^, ^4^, ^5^, ^7^, ^8^, ^15^ The compound action potentials we recorded on optical stimulation of the murine marrow cavity had latencies consistent with mostly C, not Aδ fiber activation, despite electrical stimulation of the murine marrow cavity evoking Aδ fiber activity in the same nerve. A similar finding was reported using optical vagal stimulation in the same *Wnt1‐ChR2* mice,^16^ suggesting preferential activation of C fibers by optical stimulation in *Wnt1‐ChR2* mice. However, it is likely that there are fewer Aδ fibers that innervate the bone marrow compared to C fibers, and that they have greater variance in their conduction velocities, both of which would result in less consistent compound potentials after optical stimulation. In addition, the promoter in the *Wnt1‐ChR2* mice drives ChR2 expression in all neural crest derived cells. Thus, it is not possible to be sure that the optically evoked activity we recorded from is in response to direct activation of ChR2 embedded in the membranes of bone afferent neurons, or indirect effects through activation of supporting cells. It will be important in the future to drive expression of opsins in specific subpopulations of peripheral sensory neurons, to explore their roles in bone pain more specifically.

## AUTHOR CONTRIBUTIONS

**Michael Morgan:** Conceptualization; data curation; formal analysis; funding acquisition; investigation; methodology; project administration; writing – original draft; writing – review and editing. **Hoi Ying Lee:** Data curation; formal analysis; investigation; methodology; writing – review and editing. **Aung Aung Kywe Moe:** Data curation; formal analysis; investigation; methodology; writing – review and editing. **Jenny Thai:** Data curation; formal analysis; investigation; methodology; writing – review and editing. **Jackson Hart:** Data curation; formal analysis; investigation; methodology; writing – review and editing. **Jason J. Ivanusic:** Conceptualization; data curation; formal analysis; funding acquisition; investigation; methodology; project administration; resources; writing – original draft; writing – review and editing.

## FUNDING INFORMATION

Australian National Health and Medical Research Council.

## ETHICS STATEMENT

All experiments conformed to the Australian National Health and Medical Research Council code of practice for the use of animals in research and were approved by the University of Melbourne Animal Experimentation Ethics Committee (Ethical approval number: #27597).

## CONFLICT OF INTEREST STATEMENT

The authors declare no conflicts of interest.

## Supporting information


Data S1:
