# Supplementary material for: An in vivo electrophysiological preparation for mechanical, electrical and optical stimulation of sensory neurons that innervate murine bone
Source: Animal Model Exp Med. 2025 Oct 23;8(10):1915–20. doi: 10.1002/ame2.70097 (PMC12660498; doi:10.1002/ame2.70097)
Supplement: Supplementary file 1 — Data S1: [file AME2-8-1915-s001.docx]

# **SUPPLEMENTARY METHODS**

## **Animals**

To make recordings of the activity and sensitivity of bone afferent neurons in response to mechanical, electrical and/or optical stimulation of the marrow cavity, we used a total of 40 naïve male C57BL/6 mice (8-week-old), and 4 *Wnt1-cre; ChR2-EYFP* mice (23-25-week-old). *Wnt1-cre; ChR2-EYFP* mice were generated by crossing Wnt1-cre mice (Stock No: 0022501; B6 Wnt1-Cre2; B6.Cg-E2f1^Tg(Wnt1-cre)2Sor^/J) with Ai32 (Stock No: 012569, RCL-ChR2(H134R)/EYFP) mice (Jackson Laboratories)*.* Animals were housed in groups of 2-4 in a 12-hour light/dark cycle and were provided with food and water *ad libitum*. All experiments conformed to the Australian National Health and Medical Research Council code of practice for the use of animals in research and were approved by the University of Melbourne Animal Experimentation Ethics Committee.

## **Electrophysiological preparation and recording configuration**

Experiments were performed in mice anaesthetized with isoflurane (4% induction and 1-2% maintenance; in oxygen) using a SomnoSuite Small Animal Anaesthesia System (Kent Scientific, Torrington, CT, USA). Rectal temperature was maintained within the physiological range (36-37 ˚C) with a servo-controlled heating pad (SomnoSuite, Kent Scientific). A fine branch of the nerve innervating the marrow cavity of the murine tibia was identified by careful dissection. The nerve was exposed by making a skin incision along the postero-medial aspect of the tibia and reflecting the medial head of the gastrocnemius muscle. The nerve was teased away from surrounding connective tissue and blood vessels, was cut proximally to prevent reflex activation of sympathetic efferent neurons in the nerve we recorded from, and was placed onto a platinum hook electrode for extracellular recording (Fig 1A). An indifferent electrode was implanted into a nearby muscle. The nerve was protected from desiccation by using muscle flaps to create a paraffin-filled pool maintained at room temperature. The sciatic and femoral nerves were transected high in the limb to prevent reflex activation of muscle or sympathetic efferent neurons of neurons that innervated adjacent tissues (Fig 1A).

Whole-nerve electrical activity was amplified (1000x) and filtered (high pass 100 Hz, low pass 3 kHz) using a differential amplifier (DP-311, Warner Instruments, Holliston, MA, USA), sampled at 20 kHz (Power Lab, ADInstruments, Australia) and stored to PC using LabChart recording software (LabChart 8, ADInstruments) (Fig 1A).

Mechanical stimulation was delivered by increasing intraosseous pressure within the tibial marrow cavity (Fig 1A). This was achieved by injection of heparinised (0.17 IU/mL) physiological saline (0.9% sodium chloride), delivered into the marrow cavity through a 23-gauge needle implanted into the medial surface of the proximal tibia. The needle was connected via polyethylene tubing (0.58 mm inner diameter) to a feedback-controlled syringe pump (PHD ULTRA pump, Harvard Apparatus, Holliston, MA, USA). Intraosseous pressure was measured using a bridge-amplified (TAM-D amplifier, Harvard Apparatus) signal derived from a pressure transducer (APT300 transducer, Harvard Apparatus) placed to measure the input pressure to the bone. The pump uses this as feedback to adjust flow through the system to control and maintain constant input pressures. This feature was used to deliver a ramp-and-hold mechanical stimulus with an initial flow rate of 2 ml/min during the ramp phase, and a constant pressure of 5 second duration (130, 200 or 300 mmHg) during the hold phase (Fig 1B).

Nerve impulses arising from single, mechanically or electrically activated units were discriminated from the whole-nerve recordings by their similar amplitude and duration using Spike Histogram software (LabChart 8, ADInstrument) (Fig 1B). These data were stored in parallel to PC with the whole-nerve recordings. The threshold for mechanical activation of single units was determined as the pressure at which each unit started firing, or increased firing relative to background, during the ramp phase. In some recordings, units began firing after the pressure stimulus reached the peak of the ramp-and-hold stimulus, or after the pressure ramp was turned off. We were unable to accurately record the threshold for activation of these units and so they were excluded from analysis. The discharge frequency of single units was expressed as the number of spikes per second (Hz), and was determined over the entire ramp-and-hold stimulus. To account for the effect of high frequency spontaneous activity before the onset of mechanical stimulation on our ability to accurately compare responses between repeated stimuli, discharge frequency was normalised by reporting it as the difference between a 5 second period before and during the pressure stimulus.

The conduction velocity of bone afferent neurons was determined by electrical stimulation of the marrow cavity with bipolar silver electrodes (0.5 Hz, 0.02-2 ms pulse duration, 0.05-10 V). The electrodes were placed into the marrow cavity through holes created through the outer cortex of the tibia. Waveform average analysis was used to identify spike activity that was time-locked to electrical stimulation (LabChart 8; 10-100 sweeps averaged, mean of data aligned to electrical stimulation trigger, 200 ms pre-trigger, 800 ms post-trigger). Conduction velocity was calculated by dividing the distance between the electrical stimulation site and the recording electrode, by the time taken for an action potential elicited by the electrical stimulus to reach the recording electrode. Aδ fibers were classified as those with conduction velocities between 1.4 and 13.6 m/sec, while C fibers were classified as those with conduction velocities slower than 1.4 m/sec ^21^.

To investigate how bone afferent neurons respond to repeated mechanical stimulation, pairs of 200 mmHg ramp-and-hold stimuli were delivered with a 10-minute interstimulus interval (ISI). The discharge frequency at the second stimulus was calculated as a percentage of that at the first stimulus.

To determine whether mechanically sensitive bone afferent neurons are able to code for the intensity of mechanical stimulation, we compared single unit responses to a series of pressure stimuli (130, 200 and 300 mmHg) delivered in a randomised order.

To determine if bone afferent neurons can be sensitised to mechanical stimulation, their discharge frequency and threshold for mechanical activation were assessed in response to a 200 mmHg ramp-and-hold pressure stimulus, delivered before and 30 minutes after application of capsaicin or vehicle. Due to the small size of the tibia in mice and the fragile nature of the bone, it was not possible to consistently implant a second cannula into the marrow cavity to deliver the capsaicin or vehicle as we have previously done in the rat. Instead, capsaicin (2 µM, 10 µl) or vehicle (saline, 10 µl) was delivered to the tibial marrow cavity through the same cannula as the saline used to pressurize the bone, via a second syringe attached to the tubing with a three-way tubing connector (Fig 1A).

## **Optical stimulation protocol**

To optically stimulate bone afferent neurons, *Wnt1-Cre; loxP-ChR2* and C57BL/6 mice were anaesthetised and prepared for electrophysiological recording of the nerve to the tibia as above. A 473 nm laser probe (200 μm, NA0.22, OPT_200_0.22_FLT, Doric Lenses) was placed inside the tibial marrow cavity to optically stimulate bone afferent neurons (Fig 2A and C). To insert the probe a small hole was made through the medial surface of the upper tibia, 5 mm below the knee joint line, using a 23-gauge needle. The entire optical laser probe tip was inserted 200 μm into the marrow cavity. Optical stimulation was delivered and controlled by OTPG4 software (Doric lenses) on a Windows Computer and used to trigger event markings in Labchart to accurately define stimulus-response timings. Laser power used was defined from measurements with 5 ms pulse at 1 and 10 Hz using an optical power and energy meter (PM100D, Thorlabs Inc.). Optical stimulation was also performed with the laser illuminating the nerve to the tibia before it entered the marrow cavity (Fig 2D), and on the outer surface of the cortical bone with periosteum scraped away (Fig 2E). Laser powers of 0-12.5 mWmm^-2^ were delivered at 1 Hz using the shortest functional laser pulse duration (0.25 – 0.5 ms) that could produce reliable changes in activity clearly above the baseline. Whole-nerve electrical activity was amplified (1000x) and filtered (high pass 100 Hz, low pass 3 kHz) using a differential amplifier (DP-311, Warner Instruments), sampled at 20 kHz (Power Lab, ADInstruments) and stored to PC using LabChart recording software (LabChart 8, ADInstruments). Data were converted to .Wav files and processed offline in Spike 2 (version 9, Cambridge Electronic Design, Cambridge, England). Waveform average analysis was used to identify activity time-locked to optical stimulation (LabChart 8; 100 sweep average, mean of data aligned to optical stimulation trigger, 20 ms pre-trigger, 110 ms post-trigger), with the smoothing function applied to the raw trace data. The distance between recording electrodes and the optical probe tip was measured to estimate conduction velocity. The path of the nerve from the recording site to the location of optical stimulation in the marrow cavity was not linear, so it was estimated as the distance between the recording electrode and nutrient foramen where it entered the bone, combined with the distance between the nutrient foramen and the site of optical stimulation in the tibial marrow cavity (Fig 2A).

**Statistical analyses**

Statistical analyses were performed using GraphPad Prism (version 10.4.1, Boston, MA, USA). Data are presented as mean ± Standard error of the mean. The effect of repetitive stimulation on discharge frequency, delivered at 10-minute ISIs, was assessed using paired *t-*tests. Comparisons of discharge frequencies in response to 130, 200, and 300 mmHg pressure stimuli were evaluated using one-way repeated measures ANOVA, followed by Bonferroni’s *post hoc* tests. Comparisons of single unit discharge frequency, and threshold for mechanical activation, before and 30 minutes after injection of capsaicin or vehicle control, were made with paired *t-*tests. Statistical significance was defined by *P* < 0.05. N = number of recordings, and n = number of units throughout.

**Tissue clearing and light sheet microscopy**

We used tissue clearing and light sheet microscopy to confirm expression of ChR2 in nerve terminal endings of bone afferent neurons in the marrow cavity of *Wnt1-Cre; loxP-ChR2* mouse tibia. Mice were anaesthetized (100mg/kg ketamine, 10mg/kg xylazine, i.p) and perfused via the left ventricle with 75ml 0.1M Phosphate buffered saline (PBS) followed by 75ml 4% paraformaldehyde (PFA) in 0.1M phosphate buffer. The tibiae were harvested and post-fixed in PFA at 4°C with gentle rotation for 24 hours. They were washed in 0.1M PBS (3x 10 minutes at 4°C with gentle rotation) and decalcified in Morse’s solution (22.5% formic acid, 10% tri-sodium citrate in Milli-Q H_2_O) for three nights at room temperature with gentle rotation. Unless otherwise specified, all further steps occurred at room temperature and with gentle rotation. After decalcification, samples were washed six times (15 minutes each) in Dulbecco’s phosphate buffered saline (DPBS; Sigma-Aldrich, NSW, Australia). They were gradually dehydrated in 50%, 80% and 100% methanol in DPBS (1.5 hours each), and then bleached in 6% hydrogen peroxide in methanol overnight at 4°C, protected from light and without rotation. The next day, the samples were gradually rehydrated in 100%, 100%, 80%, 50% and 0% methanol in DPBS (1.5 hours each). Each sample was blocked in DPBS containing 0.2% gelatine, 0.5% Triton X-100 and 0.01% thimerosal (DPBSG-T) for 36 hours. Samples were then immunolabeled with rabbit anti-GFP antibody (Invitrogen, #A11122, 1:500 dilution) for 11 days at 37°C with orbital rotation, washed in DPBS-T (6x 15 minutes), and then incubated in donkey anti-rabbit Alexa Fluor 647 antibody (Invitrogen, #A31573, 1:1000 dilution) for 7 days at 37°C with orbital rotation. All antibodies were diluted in DPBSG-T containing 0.1% Saponin. They were washed again in DPBS-T (6x 15 minutes), dehydrated in 20%, 40%, 60%. 80%, 100% and 100% methanol in DPBS (1 hour each), and incubated overnight in 66% dichloromethane (DCM) and 33% methanol. The following day, samples were incubated three times in DCM (30 minutes each) and then transferred to dibenzyl ether (DBE) for refractive index matching (2 hours). They were transferred to ethyl cinnamate (ECi) prior to imaging and returned to DBE for long term storage.

Cleared samples were imaged on the Ultramicroscope Blaze (Miltenyi Biotec, Bergisch Gladbach, Germany) with a Zyla sCMOS camera (Andor) and a 4x/0.35 objective lens attached to a zoom body with a magnification range of 0.6x to 2.5x. The numerical aperture of the light-sheet was 0.16, light sheet thickness was 3.91 μm and images were acquired with single-sided illumination. A 640 nm laser and a 680/30 emission filter was used. Optical z-stacks were generated using a step size of 2 µm with 100 ms exposure per step. Mosaic acquisitions were performed with 10% overlap. Images were captured using ImSpector Pro software (version 7.6.3, LaVision BioTec, Bielefeld, Germany). Images were converted to Imaris format, stitched, and visualized using the Imaris software package (version 10.2.0, Bitplane; Oxford Instrument, Abingdon, UK).
